# Using 50 K Single Nucleotide Polymorphisms to Elucidate Genomic Architecture of Line 1 Hereford Cattle

**DOI:** 10.3389/fgene.2012.00285

**Published:** 2012-12-14

**Authors:** Y. Huang, C. Maltecca, M. D. MacNeil, L. J. Alexander, W. M. Snelling, J. P. Cassady

**Affiliations:** ^1^Department of Animal Science, North Carolina State UniversityRaleigh, NC, USA; ^2^Fort Keogh Livestock and Range Research Laboratory, United States Department of Agriculture-Agricultural Research ServiceMiles City, MT, USA^‡^; ^3^United States Meat Animal Research Center, United States Department of Agriculture-Agricultural Research ServiceClay Center, NE, USA^‡^

**Keywords:** linkage disequilibrium, persistence of phase, extended haplotype homozygosity, QTL, genetic load

## Abstract

Hereford is a major beef breed in the USA, and a sub-population, known as Line 1 (L1), was established in 1934 using two paternal half-sib bulls and 50 unrelated females. L1 has since been maintained as a closed population and selected for growth to 1 year of age. Objectives were to characterize the molecular genetic architecture of L1 (*n* = 240) by comparing a cross-section of L1 with the general US. Hereford population (AHA, *n* = 311), estimating effects of imposed selection within L1 based on allele frequencies at 50 K SNP loci, and examining loci-specific effects of heterozygosity on the selection criterion. Animals were genotyped using the Illumina BovineSNP50 Beadchip, and SNP were mapped to UMD3.0 assembly of the bovine genome sequence. Average linkage disequilibrium (LD), measured by square of Pearson correlation, of adjacent SNP was 0.36 and 0.16 in L1 and AHA, respectively. Difference in LD between L1 and AHA decreased as SNP spacing increased. Persistence of phase between L1 and AHA decreased from 0.45 to 0.14 as SNP spacing increased from 50 to 5,000 kb. Extended haplotype homozygosity was greater in L1 than in AHA for 95.6% of the SNP. Knowledge of selection applied to L1 facilitated a novel approach to QTL discovery. Minor allele frequency was (FDR < 0.01) affected by cumulative selection differential at 191 out of 25,901 SNP. With the FDR relaxed to 0.05, 13 regions on BTA2, 5, 6, 9, 11, 14, 15, 18, 23, and 26 are co-located with previously identified QTL for growth. After adjustment of postweaning gain phenotypes for fixed effects and direct additive genetic effects, regression of residuals on genome-wide heterozygosity was −235.3 ± 91.6 kg. However, no SNP-specific loci where heterozygotes were significantly superior to the average of homozygotes were revealed (FDR ≥ 0.17). In conclusion, genome-wide SNP genotypes clarified effects of selection and inbreeding within L1 and differences in genomic architecture between the population segment L1 and the AHA population.

## Introduction

Hereford is a major breed of beef cattle in the United States. Over time, the Hereford breed has been subjected to a variety of different selection goals. A sub-population known as Line 1 (*L1*) was established in 1934 by joining two paternal half-sib bulls with 50 unrelated Hereford females. Thereafter, L1 has been selected primarily for growth to 1 year of age and continuously maintained as a closed population (MacNeil, 2009). Recent separation of populations can produce dramatic genomic consequences, such as different multi-locus heterozygosity in different genomic regions (Oleksyk et al., 2008). In addition, strong recent bottlenecks, such as that which occurred at the founding of L1, may cause marked founder effects (Nei et al., 1975).

Estimates of linkage disequilibrium (LD) may identify non-random associations of linked loci and may indicate population divergence (de Roos et al., 2008). Additional measures, such as extended haplotype homozygosity (EHH) may also reveal locus-specific genetic variants associated with line divergence (Tang et al., 2007). Recent studies of populations’ genomic architecture have frequently targeted regions of low heterozygosity. However, a few have identified regions of increased heterozygosity or that showed a heterozygote advantage on survival traits (Hedrick et al., 1991; Arkush et al., 2002).

The primary objective of this research was to characterize the molecular genetic architecture of Line 1 Hereford cattle using 50 K SNP; first by comparing a cross-section of L1 with the general US. Hereford population (AHA); second, by estimating effects of the imposed selection on allele frequencies and; finally by examining loci-specific effects of heterozygosity on the selection criterion.

## Materials and Methods

### Animals

Two Hereford populations were used in this research: L1 located at Fort Keogh Livestock and Range Research Laboratory, Miles City, MT and registered Herefords sampled from breeders across the US. The L1 population was founded by two paternal half-sib bulls and 50 cows in 1934 and has been selected primarily for growth to 1 year of age since that time. MacNeil (2009) reviewed use of L1 since its inception. Two hundred forty animals from L1 were genotyped for this study. They included: 57 females and 62 males (born in 2007 and 2008) from nine paternal half-sib families, and 121 ancestral sires that were born from 1953 to 2006. Although L1 is a closed population, it is related to US. Hereford due to the fact that it was generated from US. Hereford and has been influencing US. Hereford (Dickenson, 1984). To represent the general US. Hereford population registered Hereford bulls were sampled from across the US. for use in the US. Meat Animal Research Center (MARC) 2,000 bull project (Kuehn et al., 2011). Fifty-six of these bulls were previously used in the MARC Germplasm Evaluation program (http://www.ars.usda.gov/Main/docs.htm?docid=6238), while the remaining bulls (*N* = 255) were chosen by the American Hereford Association as being generally representative of the Hereford breed. A pedigree consisting of 21,284 Herefords, including 9,419 L1 Hereford cattle was used herein.

### Genotypes

All animals were genotyped using the Illumina BovineSNP50 Beadchip (Matukumalli et al., 2009). Chromosome information and physical positions of SNP were mapped to UMD3.0 assembly (Zimin et al., 2009). Genotypes were checked for inconsistencies between sire and offspring. Before applying any quality control on the SNP, out of 286 sire-offspring pairs with genotype information, 275 pairs had 5.2 ± 2.75 alternative homozygous genotypes. Eleven sire-offspring pairs had more than 1,000 alternative homozygous genotypes. These were considered as reflecting a pedigree error. When a pedigree error was found, an attempt was made to establish the correct parentage among animals with their genotypes available. Pedigree errors of six sire-offspring pairs were thus corrected and five sire-offspring pairs were considered unrelated animals. In addition, less than 50 inconsistent genotypes between sire and offspring pairs were set to missing in the offspring. With the genotypes from AHA and L1 animals combined, SNP without chromosome information and physical positions, untyped on more than 8% of animals or with a call rate less than 0.90 were removed leaving 99.9% of the original genotypes. Given the nature of the different analyses, additional edits were employed for investigations of LD, heterozygosity, integrated EHH, and association with cumulative selection differentials (CSD, detail in later text). Excluding SNP with a minor allele frequency (MAF) less than 2% in L1 yielded 35,385 SNP for LD estimation. In addition, SNP pairs in complete LD (*r*^2^ > 0.99, details in later text) had one SNP removed from the pair. Numbers of SNP used in different analyses are shown in Table 1.

**Table 1:** **Number (n) of SNP used to estimate linkage disequilibrium (LD), integrated extended haplotype homozygosity, and for regression of allele frequency on cumulative selection differential in Line 1 Hereford (L1) and the general Hereford population (AHA)**.

Analysis	Linkage disequilibrium^1^	Integrated extended haplotype homozygosity^2^	Regression^3^
	Number of SNP	Number of SNP	Number of SNP
L1	35,385	50,367	25,901
AHA	35,385	50,367	NA

*^1^ All SNP that passed quality control and had minor allele frequency (MAF) > 2% in L1 were used in estimation of LD*.

*^2^ All SNP that passed quality control were used to estimate integrated extended haplotype homozygosity*.

*^3^ All SNP that passed quality control, had MAF > 2%, and were not in LD with another SNP on the same chromosome were used in regression analysis*.

### Linkage disequilibrium and persistence of phase

To characterize L1 and AHA, LD was quantified as the square of the Pearson correlation between SNP genotypes (*r^2^*), with genotypes coded as 2, 1, or 0 to represent the number of copies of the minor allele. LD was calculated for SNP pairs in the same chromosome that had spacing between 50 to 10,000 kb. More than 91 million SNP pairs that had different spacing were used to estimate LD for L1 and AHA. Chromosome-wide LD was also estimated by using average *r*^2^ of adjacent SNP. Genome-wide average distance between adjacent SNP was 238 kb.

Genotypes were phased within each population using BEAGLE (Browning and Browning, 2007). BEAGLE forms directed acyclic graphs to perform localized haplotype phasing. Scale and shift parameters control complexity of the phasing model. These two parameters were set to 1 and 0.05, respectively, to fit the sample size and marker density in this study. Chromosomes were phased individually. Following de Roos et al. (2008), persistence of phase between L1 and AHA was calculated as the correlation of *r* across populations, where *r* is the Pearson correlation between SNP genotypes within each population, using https://www.msu.edu/~steibelj/JP_files/LD_estimate.html. Single nucleotide polymorphism pairs from 10 to 5,000 kb apart were included in this analysis.

Integrated EHH has been used to estimate the differential decay of homozygosity over physical distance. Extreme values detected by this counting algorithm have been taken to be signatures of recent selection. Here, the method of Tang et al. (2007) contrasting the EHH profiles of L1 and AHA, as quantified by In(Rsb)′, was used to further discern differences in genomic architecture between the populations.

### Regression of allele frequency on cumulative selection differential

Cumulative selection differentials (*CSD*, *n* = 7,569) for postweaning gain in L1 were calculated following MacNeil et al. (1998): for each individual, its CSD is the sum of its phenotypic deviation from its contemporary group, half of its sire’s CSD and half of its dam’s CSD. This measure reflects selection applied since the inception of the Line 1 Hereford population. To identify loci that were putatively affected by selection, the number of minor alleles at a locus was regressed on CSD for postweaning gain, one SNP at a time. It was assumed that the random residuals were distributed N (0, *I*σ_e_^2^). Two sets of putative QTL are presented from this analysis. In the first analysis, false discovery rate (*FDR*) with a critical threshold of 0.01 was employed to adjust for multiple testing (Storey, 2002). For the second set of putative QTL, the FDR was relaxed to 0.05, with the concurrent requirement that a QTL related to growth was previously identified overlapping the region. To better present results in Figure 2, if significant SNP were within 1 Mb of each other, they were arbitrarily grouped into single genomic regions.

### Heterozygosity and inbreeding

Non-additive genetic effects on postweaning gain may exist in L1, since inbreeding depression has been observed (MacNeil et al., 1992). Empirical best linear unbiased prediction (*EBLUP*) that can detect the non-additive proportion of genetic variance was employed here by modifying the method of Gulisija et al. (2007). Using ASReml (Gilmour et al., 2006), EBLUP for postweaning gain were estimated for L1 (9,419 animals in pedigree; 6,790 animals with records). The model employed was:

$$\begin{array}{rcll} y=1\mu \text{ + }X\beta \text{ + }Z\upsilon \text{ + }e & & & \end{array}$$

where *y* represents a vector of phenotypes of postweaning gain, μ represents a constant mean; *X* represents an incidence matrix of contemporary group effects including birth year, season, sex, and age of dam; β represents the regression coefficients for contemporary group effects; *Z* represents an incidence matrix of individuals; *u* represents a vector of coefficients for random additive genetic animal effects and *e* is the random residual for each observation. Additive genetic and residual effects were assumed to be independent with $u\sim N\left(0,\;A\sigma_{a}^{2}\right)$ and $e\sim N\left(0,\;I\sigma_{e}^{2}\right)$ where *A* represents the numerator relationship matrix.

The postweaning gain EBLUP residual vector $\hat{\text{e}}$ was computed as:

$$\begin{array}{rcll} \hat{\text{e}}=y-X\beta -Z\hat{\upsilon },\;\text{and} & & & \end{array}$$

was expected to be “free” of additive effects. First, the $\hat{\text{e}}$ were regressed on the pedigree-based inbreeding coefficients and genome-wide heterozygosity of the individuals. Then, to estimate the effect of genomic state (heterozygous = 1, homozygous = 0) of SNP on postweaning gain, the $\hat{\text{e}}$ were regressed on the genomic state of each individual, at each locus. A FDR of 0.05 was applied as a significance threshold.

## Results and Discussion

### Contrast of the L1 and AHA populations

For L1 and AHA genotyped animals, average inbreeding coefficients were 0.29 ± 0.022 and 0.04 ± 0.048, respectively. Effective population sizes estimated from the inter-generational change in inbreeding based on pedigree were 56.2 and 122.1 for L1 and AHA, respectively. Smaller effective population size and greater level of inbreeding in L1 were caused by the stringent bottleneck that occurred at its founding. After setting the MAF threshold at 2% (resulted in a removal of 14,777 SNP in L1 and 8,327 SNP in AHA) and pruning SNP pairs in complete LD (resulted in a further removal of 9,689 SNP in L1 and 2,379 SNP in AHA), L1 had 13,850 fewer remaining SNP than AHA.

### Linkage disequilibrium and persistence of phase

**Figure 1:**
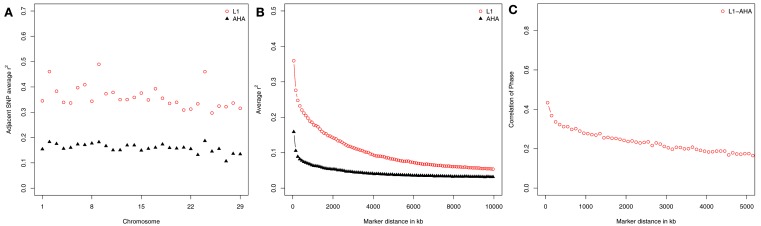
**Estimates of linkage disequilibrium (r2) across chromosomes (A) and marker spacing (B) for Line 1 Hereford (L1) and the general Hereford population (AHA), and the persistence of phase between them (C)**.

Linkage disequilibrium is influenced by effective population size and recombination rate (Tenesa et al., 2007). Therefore, LD within a population indicates characteristics of population structure (de Roos et al., 2008; Meadows et al., 2008; Qanbari et al., 2010). Average LD of adjacent SNP was 0.36 and 0.16 in L1 and AHA, respectively. Chromosome-wide average *r*^2^ of two adjacent SNP differed in different chromosomes, particularly in L1 (Figure 1A). Both L1 and AHA had the highest LD in BTA2, 9, and 24. The average *r*^2^ values in these three chromosomes were greater than 0.48 in L1, and greater than 0.18 in AHA. McKay et al. (2007) also observed a non-uniform distribution of LD across the bovine genome.

Using SNP pairs of different spacing, LD within population was estimated for L1 and for AHA (Figure 1B). Irrespective of distance between SNP, greater LD was observed in L1 than in AHA. The Line 1 Hereford population had a severe bottleneck when the population was founded, and substantial LD is generated when a population bottleneck occurs (Reich et al., 2001). As SNP spacing increased from 50 to 10,000 kb, the average *r*^2^ of SNP pairs decreased from 0.35 ± 0.005 to 0.05 ± 0.0003 in L1 and from 0.15 ± 0.004 to 0.03 ± 0.0004 in AHA. Small *r*^2^ values in long range spacing between SNP indicates large effective population size in the past while large *r*^2^ values in short range spacing between SNP indicates small effective population size in recent time (de Roos et al., 2008). Line 1 was separated from the general Hereford population in 1934. Thus, the effective population size of both populations was identical prior to that time, and the patterns of *r*^2^ reflect the recent divergence of the two populations. The rate of decrease in LD with genetic marker distance was greater in L1 than that in AHA. This indicates that the LD generated by the population bottleneck has rapidly broken down with generations. It may also reflect the current breeding practice that is designed to keep inbreeding low (MacNeil, 2009).

Persistence of phase was estimated to further quantify the relationship of L1 and AHA. It decreased from 0.45 to 0.14 as SNP spacing increased from 50 to 5,000 kb (Figure 1C). The positive correlations of phase indicate similarity across populations, again consistent with the fact that L1 was historically part of the general US. Hereford population and has continued to influence it. In 1984, 57% of the bulls listed in the American Hereford Association Sire evaluation had Line 1 ancestry (Dickenson, 1984) and as of 2010, 81% of the Hereford population in the US. had some pedigree relationship to L1 (Vicki Leesburg, USDA-ARS, Miles City, MT, USA, personal communication).

**Figure 2:**
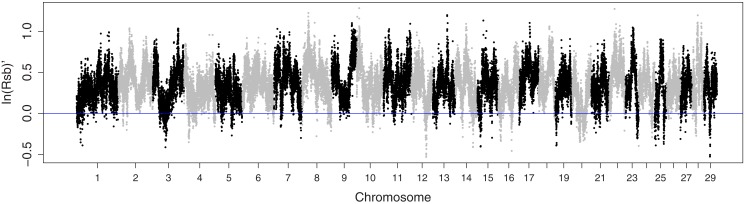
**Manhattan plot of extended haplotype homozygosity contrasting Line 1 Hereford cattle with a broader sample of the Hereford breed**.

Both L1 and AHA are known to be under selection for increased growth (MacNeil, 2009; American Hereford Association, 2012). EHH was greater in L1 than in AHA for 95.6% of the SNP (Figure 2). However, L1 confounds selection with concurrent inbreeding. Thus, the greater EHH in L1 was thought to reflect the difference between populations in inbreeding, with any signature of differential selection largely masked.

### Quantitative trait locus discovery

**Figure 3:**
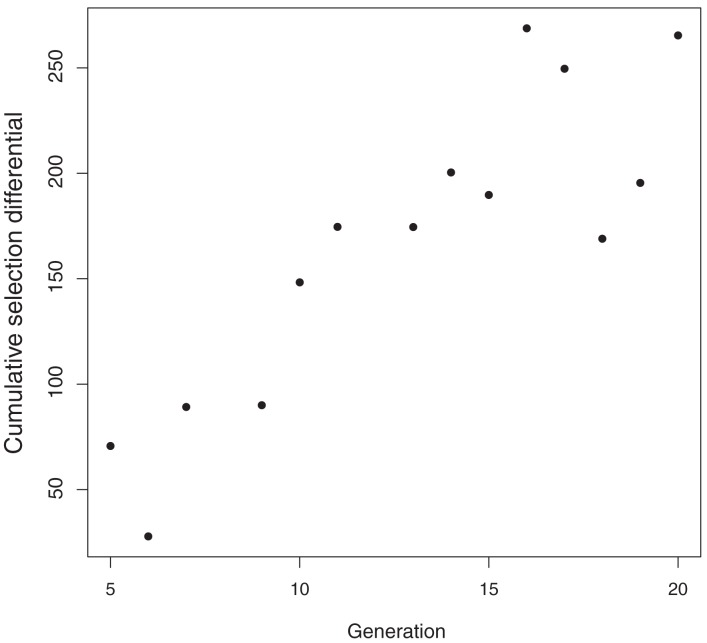
**Generation mean cumulative selection differentials for postweaning gain (kg) in Line 1 Hereford cattle**.

Allele frequency is expected to change as a result of selection (Johansson et al., 2010). CSD measure the selection applied since the inception of a selection experiment and show the expected response when heritability is 100% (Falconer and Latyszewski, 1952). Average CSD for postweaning gain per generation for genotyped L1 increased steadily (Figure 3).

In L1, MAF was significantly (FDR < 0.01) affected by CSD at 191 out of 25,901 SNP. When the FDR was relaxed to 0.05 there were 1,081 significant SNP clustered into 186 candidate regions (Table S1 in Supplementary Material). As shown in Table 2, 13 of these regions on BTA2, 5, 6, 9, 11, 14, 15, 18, 23, and 26 are co-located with previously identified QTL for traits related to growth: postweaning gain (Kneeland et al., 2004), carcass weight (Casas et al., 2000; Kim et al., 2003; Mizoshita et al., 2005; Takasuga et al., 2007; Setoguchi et al., 2009), average daily gain (Li et al., 2002, 2004; Kneeland et al., 2004; Nkrumah et al., 2007; Gutierrez-Gil et al., 2009; Marquez et al., 2009), yearling weight (Casas et al., 2000), harvest weight (Elo et al., 1999), preweaning gain (Kneeland et al., 2004), and dry matter intake (Nkrumah et al., 2007; Marquez et al., 2009; Sherman et al., 2009). For all SNP in one region, the largest regression coefficient was reported as the effect size for the region. For instance, on BTA2 in the interval from 10.6 to 11.5 Mb, the regression of MAF on CSD (kg × 100) was 0.14 ± 0.034 and this interval is coincident with previously discovered QTL for postweaning gain and carcass weight (Kim et al., 2003; Kneeland et al., 2004).

**Table 2:** **Genomic regions wherein the regression (b ± SE) of number of minor alleles on cumulative selection differential for postweaning gain was significant (FDR < 0.05) in L1 and overlapped with previously identified QTL**.

BTA	Locus (Mb)	b ± SE^1^	Traits^2^	Reference
2	10.6–11.5	0.14 ± 0.03	CW, PWG	Kim et al. (2003), Kneeland et al. (2004)
5	56.7–57.4	0.16 ± 0.03	ADG	Li et al. (2002), Li et al. (2004)
6	39.6–42.0	0.20 ± 0.04	YW, CW	Casas et al. (2000), Setoguchi et al. (2009)
6	52.4–63.2	0.21 ± 0.04	GBW, ADG	Kneeland et al. (2004)
9	14.9–16.0	0.18 ± 0.05	ADG	Marquez et al. (2009)
11	18.7–23.8	0.24 ± 0.05	DMI	Marquez et al. (2009)
14	19.8–23.6	0.15 ± 0.05	PWG, CW	Kneeland et al. (2004), Mizoshita et al. (2005), Takasuga et al. (2007)
15	38.0–39.9	0.19 ± 0.05	ADG	Marquez et al. (2009)
18	37.3–40.5	0.16 ± 0.04	DMI	Nkrumah et al. (2007)
23	19.2–20.2	0.16 ± 0.05	GBW, HW	Elo et al. (1999), Kneeland et al. (2004)
23	48.7–49.0	0.10 ± 0.03	DMI	Sherman et al. (2009)
26	27.9–32.5	0.19 ± 0.059	ADG	Gutierrez-Gil et al. (2009)
26	45.5–47.9	0.15 ± 0.05	ADG, DMI	Nkrumah et al. (2007)

*^1^ The maximum regression coefficient for number of SNP minor alleles (0, 1, 2) at a locus on CSD (kg × 100) for postweaning gain*.

*^2^ ADG, Average daily gain; CW, Carcass weight; DMI, dry matter intake; PWG, Postweaning gain; GBW, Preweaning gain; HW, Harvest weight; YW, 365−d weight*.

### Heterozygosity and inbreeding effect on postweaning gain

The regression of EBLUP residuals for postweaning gain on inbreeding coefficients (decimal) was −349.2 ± 68.2 kg. This is consistent with previous results from L1 where the regression was estimated simultaneously with the other fixed effects (MacNeil et al., 1992). Inbreeding coefficients are expectations of alleles being identical by decent estimated using pedigree information. Alternatively, inbreeding can be obtained from heterozygosity under probability theory (Kempthorne, 1969). The percentage of homozygosity of an individual can be described as a combination of identical by decent and identical in state alleles. In these data, the correlation between the inbreeding coefficients and genome-wide heterozygosity was −0.34 and the regression of EBLUP residuals for postweaning gain on genome-wide heterozygosity was −235.3 ± 91.6 kg. In this case, the regressions of EBLUP residuals for postweaning gain on inbreeding and heterozygosity indicate dominance effects on postweaning gain. Despite these significant indicators of effects of heterozygosity on postweaning gain, no significant SNP-specific effects were observed (FDR ≥ 0.17). It seems plausible that inbreeding depression results from deleterious alleles of both large and small effects (Charlesworth and Charlesworth, 1999; Wang et al., 1999) and that successive generations of inbreeding coupled with selection may purge alleles with large effect, while the genetic load resulting from mildly deleterious alleles persists (Hedrick, 1994; Wang et al., 1999). Thus, prior generations of inbreeding in L1 coupled with selection for postweaning gain may have purged the major deleterious recessive alleles, hence reducing the magnitude of inbreeding depression and making smaller locus-specific effects difficult to detect. Experimental results (e.g., MacNeil et al., 1984; and reviewed by Crnokrak and Barrett, 2002) partially support this contention.

## Conclusion

Genome-wide SNP genotypes from L1 and AHA individuals clarified relationships between the population segment of L1 and the general US. Hereford population (AHA). L1 had greater average LD than AHA, indicated by higher correlation of SNP genotypes within L1 and fewer haplotypes spanning the genome. The difference in LD between L1 and AHA decreased as SNP spacing increased, indicating their relatively recent divergence. Persistence of phase indicated that the large LD generated by the bottleneck which occurred at the formation of L1 has eroded. Knowledge of selection applied to L1 since its inception provided an opportunity for a novel approach to QTL discovery. Coupled with previous reports of QTL for growth traits in beef cattle, this approach provided 13 candidate regions for further investigation. These data also reveal no loci affecting postweaning growth where the heterozygotes were significantly superior to the average of the homozygotes.

## Conflict of Interest Statement

The authors declare that the research was conducted in the absence of any commercial or financial relationships that could be construed as a potential conflict of interest.

## Supplementary Material

The Supplementary Material for this article can be found online at http://www.frontiersin.org/Livestock_Genomics/10.3389/fgene.2012.00285/abstract fgene.2012.00285/abstract

Supplementary Table S1 **Genomic regions wherein the regression (b ± SE) of number of minor alleles on cumulative selection differential for postweaning gain was significant (FDR < 0.05) in L1**.
