# Supplementary material for: Using 50 K Single Nucleotide Polymorphisms to Elucidate Genomic Architecture of Line 1 Hereford Cattle
Source: Front Genet. 2012 Dec 14;3:285. doi: 10.3389/fgene.2012.00285 (PMC3571696; doi:10.3389/fgene.2012.00285)
Supplement: Supplementary Table S1 — Genomic regions wherein the regression (b ± SE) of number of minor alleles on cumulative selection differential for postweaning gain was significant (FDR < 0.05) in L1. [file 36017_Cassady_DataSheet1.DOCX]

**Supplement Table.** Genomic regions wherein the regression (b ± SE) of number of minor alleles on cumulative selection differential for postweaning gain was significant (FDR < 0.05) in L1

| BTA | Locus (Mb) | b ± SE^1^ | SNP^2^ | Trait^3^ | References |
| --- | --- | --- | --- | --- | --- |
| 1 | 31.7 - 34.4 | 0.22 ± 0.049 | BTB-01843131, ARS-BFGL-NGS-80747, BTB-01955576, ARS-BFGL-NGS-64530, Hapmap52004-BTA-69178, Hapmap48855-BTA-69221, BTA-91456-no-rs, BTB-00930093, Hapmap43823-BTA-27227, UA-IFASA-4161 |  |  |
| 1 | 36 | 0.2 ± 0.051 | BTB-00017988 |  |  |
| 1 | 37.3 - 39.2 | 0.16 ± 0.052 | ARS-BFGL-NGS-12168, ARS-BFGL-BAC-14429, BTA-16749-no-rs, BTA-109574-no-rs, ARS-BFGL-BAC-11946 |  |  |
| 1 | 48.1 | 0.14 ± 0.048 | ARS-BFGL-NGS-73983 |  |  |
| 1 | 50.9 | 0.16 ± 0.052 | Hapmap41716-BTA-100042 |  |  |
| 1 | 64 | 0.1 ± 0.026 | ARS-BFGL-NGS-105436 |  |  |
| 1 | 66.8 | 0.07 ± 0.021 | Hapmap54041-ss46526276 |  |  |
| 1 | 79.9 | 0.11 ± 0.035 | ARS-BFGL-NGS-44036, Hapmap51438-BTA-37566 |  |  |
| 1 | 85.5 | 0.09 ± 0.028 | Hapmap33711-BTA-124326 |  |  |
| 1 | 88.6 | 0.11 ± 0.036 | BTA-40510-no-rs |  |  |
| 1 | 125.9 | 0.11 ± 0.035 | BTB-01369713 |  |  |
| 1 | 133 - 134.8 | 0.17 ± 0.052 | BTB-00059569, ARS-BFGL-NGS-44780, Hapmap24138-BTA-124742, BTA-53322-no-rs, BTB-01789695, ARS-BFGL-NGS-3162 |  |  |
| 2 | 10.6 - 11.5 | 0.14 ± 0.034 | Hapmap44041-BTA-23382, ARS-BFGL-NGS-53911 | CW, PWG | Kim et al., 2003; Kneeland et al., 2004 |
| 2 | 43.5 - 43.9 | 0.16 ± 0.048 | ARS-BFGL-NGS-8309, ARS-BFGL-NGS-79459 |  |  |
| 2 | 45.3 - 46.8 | 0.21 ± 0.047 | ARS-BFGL-NGS-27169, Hapmap53891-rs29021231, Hapmap34848-BES1_Contig523_1341, ARS-BFGL-BAC-41571, Hapmap50824-BTA-92238, Hapmap44600-BTA-47582 |  |  |
| 2 | 84.2 | 0.15 ± 0.048 | Hapmap26980-BTA-149220 |  |  |
| 2 | 133.7 | 0.14 ± 0.043 | ARS-BFGL-NGS-22911 |  |  |
| 3 | 6.7 - 6.8 | 0.17 ± 0.047 | Hapmap42294-BTA-69421, ARS-BFGL-NGS-113472 |  |  |
| 3 | 15.8 - 16.1 | 0.15 ± 0.041 | ARS-BFGL-NGS-54121, Hapmap39423-BTA-69854, ARS-BFGL-NGS-37429 |  |  |
| 3 | 27.1 | 0.07 ± 0.022 | ARS-BFGL-NGS-42733 |  |  |
| 3 | 57.7 - 58.4 | 0.2 ± 0.048 | INRA-353, Hapmap47376-BTA-67990, Hapmap55448-rs29020629, BTA-16282-no-rs, ARS-BFGL-NGS-20652 |  |  |
| 3 | 60 - 62.2 | 0.16 ± 0.051 | BTB-02020352, BTB-00130969, BTB-01322824, BTA-22681-no-rs, BTB-00130588, BTB-01660862, Hapmap48047-BTA-68130, ARS-BFGL-NGS-112148, BTB-01982674 |  |  |
| 3 | 63.5 - 63.9 | 0.11 ± 0.033 | BTA-96764-no-rs, Hapmap35621-SCAFFOLD36250_11008 |  |  |
| 3 | 66.3 - 67 | 0.17 ± 0.044 | Hapmap50576-BTA-114951, BTB-01203398, BTB-01203844 |  |  |
| 3 | 68.4 - 69.6 | 0.14 ± 0.043 | ARS-BFGL-NGS-5902, BTB-00134027, INRA-99, BTB-00134789, BTB-00134756 |  |  |
| 3 | 70.6 - 72.1 | 0.17 ± 0.047 | UA-IFASA-998, BTA-19340-no-rs, BTB-01093811, ARS-BFGL-NGS-8303, BTB-01281916, Hapmap27208-BTA-157501, BTB-00135233, BTB-01281672 |  |  |
| 3 | 73.2 | 0.12 ± 0.04 | Hapmap42603-BTA-61004 |  |  |
| 3 | 74.2 | 0.17 ± 0.044 | Hapmap57979-rs29017982 |  |  |
| 3 | 75.2 | 0.15 ± 0.047 | BTA-68347-no-rs |  |  |
| 3 | 76.4 | 0.15 ± 0.047 | BTB-02041459 |  |  |
| 3 | 77.5 - 78.1 | 0.17 ± 0.048 | ARS-BFGL-NGS-39184, BTA-68406-no-rs, BTA-115703-no-rs, ARS-BFGL-NGS-33377 |  |  |
| 3 | 79.3 - 80.3 | 0.2 ± 0.048 | BTB-01155381, ARS-BFGL-NGS-60582, ARS-BFGL-NGS-31953, Hapmap28134-BTA-152098, ARS-BFGL-NGS-118303 |  |  |
| 3 | 81.6 - 82.3 | 0.2 ± 0.048 | Hapmap33205-BTA-141538, Hapmap34665-BES10_Contig789_3212, Hapmap58865-ss46526158 |  |  |
| 3 | 83.8 - 87.2 | 0.2 ± 0.045 | ARS-BFGL-NGS-17040, BTA-29968-no-rs, ARS-BFGL-NGS-96076, BTB-01466258, BTA-95406-no-rs, Hapmap34100-BES3_Contig74_659, Hapmap39464-BTA-116273, Hapmap43465-BTA-111846, Hapmap47785-BTA-95420 |  |  |
| 3 | 88.7 - 90.3 | 0.22 ± 0.051 | BTB-00141992, ARS-BFGL-NGS-104839, ARS-BFGL-NGS-80560, BTB-00141843, BTB-01893977, Hapmap41488-BTA-24106, BTB-00143361, BTB-00143286, BTB-00143272, BTB-00142718, ARS-BFGL-NGS-112680, BTB-00142582, ARS-BFGL-NGS-33677, Hapmap34238-BES1_Contig417_1330 |  |  |
| 3 | 93.3 - 93.7 | 0.17 ± 0.051 | Hapmap42636-BTA-68725, UA-IFASA-2729, BTB-00144361, Hapmap23711-BTA-31541 |  |  |
| 3 | 102.6 - 103.4 | 0.18 ± 0.043 | ARS-BFGL-NGS-32606, ARS-BFGL-NGS-20122, ARS-BFGL-NGS-118597, Hapmap48854-BTA-69129 |  |  |
| 3 | 118.1 | 0.17 ± 0.05 | ARS-BFGL-NGS-90439 |  |  |
| 4 | 4.6 - 5.5 | 0.15 ± 0.044 | Hapmap38452-BTA-98747, ARS-BFGL-NGS-41865, Hapmap27594-BTA-141889, Hapmap40914-BTA-122270 |  |  |
| 4 | 9.2 | 0.15 ± 0.046 | Hapmap39581-BTA-70101 |  |  |
| 4 | 28.1 | 0.11 ± 0.036 | Hapmap44790-BTA-87385 |  |  |
| 4 | 29.5 - 30.8 | 0.22 ± 0.047 | BTB-01202801, ARS-BFGL-NGS-14672, ARS-BFGL-NGS-63028, ARS-BFGL-NGS-70466 |  |  |
| 4 | 36.2 | 0.09 ± 0.03 | Hapmap43100-BTA-92592 |  |  |
| 4 | 53.4 - 53.7 | 0.15 ± 0.047 | ARS-BFGL-NGS-38893, Hapmap45967-BTA-99756, Hapmap50535-BTA-99757 |  |  |
| 4 | 56.2 | 0.11 ± 0.035 | BTA-70675-no-rs |  |  |
| 4 | 59.1 | 0.09 ± 0.028 | Hapmap43661-BTA-70769 |  |  |
| 4 | 77.6 | 0.16 ± 0.048 | ARS-BFGL-NGS-33546 |  |  |
| 4 | 80.4 | 0.18 ± 0.047 | Hapmap42298-BTA-71527, BTB-00200250 |  |  |
| 4 | 81.4 - 82.6 | 0.15 ± 0.048 | BTB-00201173, BTB-00566951, BTB-00922140 |  |  |
| 4 | 84.9 | 0.14 ± 0.048 | BTB-02023591 |  |  |
| 4 | 97.4 - 98.2 | 0.17 ± 0.052 | Hapmap50765-BTA-71773, Hapmap50074-BTA-71803 |  |  |
| 4 | 109.9 - 110.4 | 0.16 ± 0.049 | ARS-BFGL-NGS-1972, Hapmap48062-BTA-72409 |  |  |
| 4 | 111.4 - 112.7 | 0.16 ± 0.043 | Hapmap33207-BTA-142657, Hapmap34027-BES1_Contig459_655, ARS-BFGL-NGS-111592, ARS-BFGL-NGS-19306 |  |  |
| 5 | 0.4 - 1.2 | 0.17 ± 0.05 | BTB-01711382, BTB-01735789, BTB-01735758, BTB-01089317 |  |  |
| 5 | 4.7 | 0.16 ± 0.048 | BTA-29483-no-rs |  |  |
| 5 | 7 | 0.17 ± 0.039 | ARS-BFGL-NGS-118379 |  |  |
| 5 | 9.7 | 0.14 ± 0.044 | Hapmap58252-rs29024363 |  |  |
| 5 | 23.6 - 26.1 | 0.18 ± 0.044 | BTA-72947-no-rs, ARS-BFGL-NGS-100276, ARS-BFGL-NGS-77906, BTA-73025-no-rs, Hapmap31455-BTA-148845, Hapmap41950-BTA-72999 |  |  |
| 5 | 28.9 | 0.14 ± 0.046 | ARS-USMARC-624 |  |  |
| 5 | 37.4 - 37.5 | 0.18 ± 0.048 | BTB-01248388, BTA-118501-no-rs |  |  |
| 5 | 45.1 - 45.7 | 0.19 ± 0.045 | BTA-106792-no-rs, Hapmap41954-BTA-73479, ARS-BFGL-NGS-42961, ARS-BFGL-NGS-109171 |  |  |
| 5 | 47 - 47.1 | 0.19 ± 0.049 | Hapmap50837-BTA-98392, BTA-98387-no-rs |  |  |
| 5 | 51.1 | 0.09 ± 0.029 | ARS-BFGL-NGS-33745 |  |  |
| 5 | 55.3 - 55.4 | 0.16 ± 0.037 | BTA-54940-no-rs, BTA-54938-no-rs, ARS-BFGL-NGS-92026 |  |  |
| 5 | 56.7 - 57.4 | 0.16 ± 0.033 | Hapmap30258-BTA-143119, UA-IFASA-6196, Hapmap33935-BES11_Contig132_791 | ADG | Li et al., 2002; Li et al., 2004 |
| 5 | 60.4 - 60.7 | 0.13 ± 0.029 | Hapmap53993-rs29024740, ARS-BFGL-NGS-35987 |  |  |
| 5 | 64.1 | 0.12 ± 0.039 | ARS-BFGL-NGS-60284 |  |  |
| 5 | 70.2 | 0.08 ± 0.023 | BTA-73842-no-rs |  |  |
| 5 | 71.9 - 72.3 | 0.13 ± 0.043 | ARS-BFGL-NGS-111008, Hapmap40191-BTA-73919, ARS-BFGL-NGS-111053 |  |  |
| 5 | 86.3 | 0.08 ± 0.023 | BTA-123014-no-rs, Hapmap29308-BTA-143360 |  |  |
| 5 | 88.6 | 0.14 ± 0.045 | BTB-00234287 |  |  |
| 5 | 115.1 - 115.7 | 0.15 ± 0.043 | ARS-BFGL-NGS-111201, BTA-75208-no-rs, Hapmap41340-BTA-71012 |  |  |
| 5 | 120.6 | 0.09 ± 0.028 | ARS-BFGL-NGS-110517 |  |  |
| 6 | 16.2 | 0.11 ± 0.038 | BTB-00247173 |  |  |
| 6 | 33.2 | 0.15 ± 0.051 | Hapmap27069-BTC-032712 |  |  |
| 6 | 34.9 | 0.16 ± 0.048 | ARS-BFGL-NGS-103009 |  |  |
| 6 | 35.9 - 36.7 | 0.14 ± 0.04 | ARS-BFGL-NGS-114855, ARS-BFGL-NGS-49600, Hapmap27329-BTC-055310 |  |  |
| 6 | 38 - 38.2 | 0.13 ± 0.031 | BTA-22850-no-rs, Hapmap27503-BTC-033786 |  |  |
| 6 | 39.6 - 42 | 0.2 ± 0.044 | Hapmap26618-BTC-070864, Hapmap33742-BTC-041190, Hapmap27701-BTC-050761, ARS-BFGL-NGS-34023, Hapmap57625-rs29027071, Hapmap48459-BTA-75920 | YW, CW | Casas et al., 2000; Setoguchi et al., 2009 |
| 6 | 45.1 - 46.8 | 0.19 ± 0.045 | Hapmap31283-BTC-039096, Hapmap44618-BTA-76049, ARS-BFGL-NGS-97136, Hapmap55150-rs29025709, Hapmap23226-BTA-159656, Hapmap52362-ss46526804, ARS-BFGL-NGS-95035 |  |  |
| 6 | 52.4 - 55.4 | 0.21 ± 0.042 | Hapmap55435-rs29009844, Hapmap23860-BTC-065677, BTA-86872-no-rs, ARS-BFGL-NGS-2697, ARS-BFGL-NGS-23616 | GBW, ADG | Kneeland et al., 2004 |
| 6 | 56.4 - 57.2 | 0.16 ± 0.035 | ARS-BFGL-NGS-27268, ARS-BFGL-NGS-28430, Hapmap54176-rs29021880 |  |  |
| 6 | 58.3 - 63.2 | 0.21 ± 0.042 | Hapmap51047-BTA-76410, BTA-21386-no-rs, Hapmap42666-BTA-76393, ARS-BFGL-NGS-109509, Hapmap58166-rs29026792, ARS-BFGL-NGS-31848, ARS-BFGL-NGS-34791, BTA-97850-no-rs, BTA-97854-no-rs, Hapmap55170-rs29017690, ARS-BFGL-NGS-101874, Hapmap51979-BTA-43275, ARS-BFGL-NGS-62682, BTA-30686-no-rs, Hapmap51763-BTA-76571, ARS-BFGL-NGS-11358, BTB-00248845, ARS-BFGL-NGS-111961, ARS-BFGL-NGS-117527, BTB-00259582, ARS-BFGL-NGS-1852, BTB-01211574, ARS-BFGL-NGS-69759 |  |  |
| 6 | 73.9 - 74.5 | 0.13 ± 0.043 | ARS-BFGL-NGS-69314, BTB-00263615, Hapmap50462-BTA-76734 |  |  |
| 6 | 75.7 - 76.1 | 0.16 ± 0.041 | Hapmap57992-rs29022820, Hapmap51154-BTA-115998, Hapmap55589-rs29013237 |  |  |
| 6 | 80.8 | 0.11 ± 0.038 | BTB-01428552 |  |  |
| 6 | 88.5 | 0.16 ± 0.051 | BTA-10392-no-rs |  |  |
| 6 | 89.6 - 90.7 | 0.15 ± 0.051 | BTB-01946648, BTA-121769-no-rs, Hapmap42616-BTA-64032, Hapmap43045-BTA-76998 |  |  |
| 6 | 91.8 - 94.6 | 0.2 ± 0.049 | BTA-86242-no-rs, ARS-BFGL-NGS-23066, ARS-BFGL-NGS-40151, ARS-BFGL-NGS-63919, ARS-BFGL-NGS-92582, BTB-00267864, ARS-BFGL-NGS-63000, ARS-BFGL-NGS-24779, ARS-BFGL-NGS-41209 |  |  |
| 6 | 95.7 - 96 | 0.22 ± 0.049 | ARS-BFGL-NGS-118552, ARS-BFGL-NGS-29722, Hapmap41966-BTA-77379, BTA-77394-no-rs, BTB-00272142 |  |  |
| 6 | 97.1 - 99.8 | 0.23 ± 0.049 | ARS-BFGL-NGS-117220, BTB-00272793, BTB-00272812, BTB-00272865, Hapmap48889-BTA-77486, ARS-BFGL-NGS-82167, Hapmap54521-ss46526011, BTB-01096206, Hapmap31859-BTA-21893 |  |  |
| 6 | 101.1 - 102.2 | 0.07 ± 0.024 | BTB-00274746, ARS-BFGL-NGS-115828, Hapmap23911-BTC-045488, Hapmap32226-BTC-045029, Hapmap32224-BTC-044678 |  |  |
| 6 | 107.8 - 108.5 | 0.16 ± 0.04 | ARS-BFGL-NGS-72514, ARS-BFGL-NGS-34909, ARS-BFGL-NGS-106770 |  |  |
| 6 | 109.9 | 0.16 ± 0.044 | ARS-BFGL-NGS-110156 |  |  |
| 6 | 112.8 | 0.12 ± 0.041 | BTA-95733-no-rs |  |  |
| 6 | 115.6 | 0.09 ± 0.03 | ARS-BFGL-NGS-100900 |  |  |
| 7 | 26.8 - 29.3 | 0.19 ± 0.05 | ARS-BFGL-NGS-104332, ARS-BFGL-NGS-30757, ARS-BFGL-NGS-109381, BTB-01074446, BTA-78695-no-rs, ARS-BFGL-NGS-18240, BTB-01153707, BTB-01154003, Hapmap33975-BES10_Contig464_714, ARS-BFGL-NGS-88212 |  |  |
| 7 | 31.2 | 0.16 ± 0.045 | ARS-BFGL-NGS-98924 |  |  |
| 7 | 32.6 - 32.7 | 0.17 ± 0.049 | ARS-BFGL-NGS-64145, Hapmap57685-rs29016545 |  |  |
| 7 | 40.9 - 41.1 | 0.16 ± 0.049 | BTB-01651805, Hapmap41157-BTA-113254 |  |  |
| 7 | 46.2 - 48.7 | 0.18 ± 0.045 | Hapmap42327-BTA-78943, UA-IFASA-4023, BTB-00309317, ARS-BFGL-NGS-20497, ARS-BFGL-NGS-45683, Hapmap34406-BES2_Contig344_1026, ARS-BFGL-NGS-24225, ARS-BFGL-NGS-76390, BTB-01860974, BTB-00310653, ARS-BFGL-NGS-79199, ARS-BFGL-NGS-110899, BTB-00022719 |  |  |
| 7 | 64.3 - 64.5 | 0.15 ± 0.036 | ARS-BFGL-NGS-104130, BTB-00315902, ARS-BFGL-NGS-84686 |  |  |
| 7 | 78.9 - 80.6 | 0.14 ± 0.041 | BTB-01339323, ARS-BFGL-NGS-176, UA-IFASA-8448, BTB-01273515, ARS-BFGL-NGS-110279, ARS-BFGL-NGS-2181, BTB-00320041 |  |  |
| 7 | 101.5 - 106.2 | 0.19 ± 0.049 | ARS-BFGL-NGS-111842, Hapmap38264-BTA-96587, BTA-45431-no-rs, Hapmap22951-BTA-163311, Hapmap43948-BTA-80452, BTA-80476-no-rs, BTB-00956256, ARS-BFGL-NGS-25018, BTB-01472249 |  |  |
| 7 | 109.2 | 0.24 ± 0.05 | ARS-BFGL-NGS-43318 |  |  |
| 7 | 111.7 - 111.8 | 0.15 ± 0.047 | ARS-BFGL-NGS-43521, BTB-00080644 |  |  |
| 8 | 5.8 - 6 | 0.08 ± 0.024 | BTB-00334209, ARS-BFGL-NGS-104101 |  |  |
| 8 | 7.2 - 7.5 | 0.07 ± 0.019 | BTA-81086-no-rs, ARS-BFGL-NGS-29411 |  |  |
| 8 | 37 | 0.08 ± 0.027 | Hapmap48192-BTA-114766 |  |  |
| 8 | 41.1 - 42.4 | 0.12 ± 0.035 | BTB-00345076, BTB-00342741, Hapmap52331-rs29021338, BTB-01581546 |  |  |
| 8 | 45 | 0.15 ± 0.037 | BTB-00344647, ARS-BFGL-NGS-81126 |  |  |
| 8 | 53.8 - 53.9 | 0.19 ± 0.052 | Hapmap49589-BTA-38292, Hapmap50345-BTA-38288 |  |  |
| 8 | 67.6 | 0.11 ± 0.036 | UA-IFASA-2124 |  |  |
| 8 | 91.8 - 95.7 | 0.19 ± 0.043 | BTA-82231-no-rs, BTB-01415906, Hapmap38258-BTA-82187, Hapmap27238-BTA-163742, BTB-00368041, BTB-00368434, Hapmap50914-BTA-21089, BTB-01670082, BTB-01266301, BTB-01266363 |  |  |
| 8 | 97 - 98 | 0.16 ± 0.05 | BTB-01435179, BTB-01067452, Hapmap50544-BTA-102614, BTA-102583-no-rs |  |  |
| 8 | 102.1 - 102.8 | 0.16 ± 0.051 | BTB-00371898, BTB-00372642 |  |  |
| 8 | 105.7 | 0.15 ± 0.044 | BTB-01401013 |  |  |
| 8 | 111.5 | 0.05 ± 0.015 | Hapmap33843-BES9_Contig546_2551 |  |  |
| 8 | 113.2 | 0.14 ± 0.045 | ARS-BFGL-NGS-106379 |  |  |
| 9 | 0.5 - 3 | 0.15 ± 0.042 | ARS-BFGL-NGS-22958, BTB-01126890, BTB-00478134, BTB-01957423, BTB-01752812, BTB-01374425, ARS-BFGL-NGS-104062, BTB-01573029, BTB-01572761, BTB-00378581 |  |  |
| 9 | 6.7 | 0.08 ± 0.028 | ARS-BFGL-NGS-103775 |  |  |
| 9 | 14.9 - 16 | 0.18 ± 0.045 | UA-IFASA-5793, BTA-85265-no-rs, ARS-BFGL-NGS-116510, Hapmap27634-BTA-158717, ARS-BFGL-NGS-105703, BTB-01643298, BTB-00380606, BTB-00380899 | ADG | Kneeland et al., 2004 |
| 9 | 32.4 - 35.3 | 0.17 ± 0.048 | ARS-BFGL-NGS-26381, BTB-00388602, BTA-83267-no-rs, BTB-00384830, BTA-83227-no-rs, BTA-18520-no-rs, BTB-00389059, BTB-00389315 |  |  |
| 9 | 58.8 | 0.09 ± 0.03 | Hapmap40011-BTA-59710 |  |  |
| 9 | 61.5 - 61.8 | 0.13 ± 0.037 | BTB-00397151, ARS-BFGL-NGS-12541 |  |  |
| 9 | 75.8 | 0.14 ± 0.046 | Hapmap51348-BTA-90742 |  |  |
| 10 | 21.2 - 22.2 | 0.18 ± 0.048 | ARS-BFGL-NGS-13835, ARS-BFGL-NGS-19822, ARS-BFGL-BAC-11616 |  |  |
| 10 | 35.2 | 0.12 ± 0.037 | Hapmap40785-BTA-66290 |  |  |
| 10 | 68.5 | 0.16 ± 0.05 | ARS-BFGL-NGS-97889 |  |  |
| 10 | 75.6 | 0.14 ± 0.04 | ARS-BFGL-NGS-105188 |  |  |
| 10 | 79.9 - 81.6 | 0.18 ± 0.05 | BTA-76288-no-rs, ARS-BFGL-NGS-41880, ARS-BFGL-BAC-11007, ARS-BFGL-NGS-73354, ARS-BFGL-NGS-113766, ARS-USMARC-Parent-DQ786762-rs29010772 |  |  |
| 10 | 87.7 - 89.6 | 0.16 ± 0.05 | Hapmap60876-rs29013997, BTB-00439466, Hapmap39978-BTA-15580, Hapmap46117-BTA-106518, ARS-BFGL-NGS-18814, ARS-BFGL-NGS-80270, BTB-00442962, ARS-BFGL-NGS-31962 |  |  |
| 10 | 93.7 - 96.2 | 0.2 ± 0.051 | ARS-BFGL-NGS-100479, BTB-00445660, ARS-BFGL-NGS-74928, BTB-00446095, BTB-00446145, BTB-01402723, BTB-01526503, BTB-01010697, BTA-15744-no-rs, BTB-01825498, BTB-01147467 |  |  |
| 10 | 103.6 | 0.11 ± 0.035 | Hapmap49930-BTA-24651 |  |  |
| 11 | 16.8 - 17.5 | 0.19 ± 0.05 | BTB-01347992, BTB-01347970, Hapmap43078-BTA-84358, BTB-01764269, Hapmap26417-BTA-147042, BTB-01766447 |  |  |
| 11 | 18.7 - 23.8 | 0.24 ± 0.049 | BTA-118292-no-rs, BTB-01396675, Hapmap25656-BTA-88183, Hapmap42709-BTA-86558, ARS-BFGL-NGS-3421, ARS-BFGL-NGS-17016, Hapmap32683-BTA-88615, Hapmap55558-rs29013980, BTB-00467628, Hapmap26194-BTA-158111, ARS-BFGL-NGS-104435, BTB-00464946, BTB-00464926, BTB-00464454, ARS-BFGL-NGS-2977, UA-IFASA-4213, BTB-00465155, BTB-00465430, BTA-87562-no-rs, ARS-BFGL-BAC-11050, Hapmap52352-rs29018193, BTA-97226-no-rs, BTA-97221-no-rs, ARS-BFGL-NGS-110642, ARS-BFGL-NGS-116483, BTB-00466422, BTB-01903909, ARS-BFGL-BAC-11672, BTB-01859163, ARS-BFGL-NGS-107853, ARS-BFGL-NGS-40621, BTA-93788-no-rs, Hapmap59290-rs29022016, Hapmap43993-BTA-103701, BTB-01444917, BTB-01444868, BTB-01444756 | DMI | Marquez et al., 2009 |
| 11 | 26.2 - 29.5 | 0.18 ± 0.05 | Hapmap54800-rs29014906, ARS-BFGL-NGS-36356, ARS-BFGL-NGS-114146, BTB-01260153, BTB-01260228, BTB-01260337, ARS-BFGL-NGS-75483, Hapmap42722-BTA-90867, ARS-BFGL-NGS-111484, BTB-00470051, ARS-BFGL-NGS-92888, ARS-BFGL-NGS-114530, ARS-BFGL-NGS-112640, ARS-BFGL-NGS-5463 |  |  |
| 11 | 32.6 | 0.1 ± 0.031 | BTA-88672-no-rs |  |  |
| 11 | 89.3 - 92.9 | 0.21 ± 0.049 | ARS-BFGL-NGS-28030, ARS-BFGL-NGS-117441, Hapmap58113-rs29014432, ARS-BFGL-NGS-61730, ARS-BFGL-NGS-92082, BTA-118584-no-rs, BTA-118582-no-rs, Hapmap30052-BTA-161211, ARS-BFGL-NGS-36344, BTA-21473-no-rs, ARS-BFGL-NGS-12870, ARS-BFGL-NGS-32722, ARS-BFGL-NGS-76359 |  |  |
| 11 | 94.8 | 0.1 ± 0.032 | ARS-BFGL-NGS-118335 |  |  |
| 11 | 97.1 - 98.5 | 0.24 ± 0.051 | ARS-BFGL-NGS-21607, ARS-BFGL-NGS-36373, ARS-BFGL-NGS-26030, ARS-BFGL-NGS-107846, ARS-BFGL-NGS-27581, ARS-BFGL-NGS-70509 |  |  |
| 11 | 99.5 - 100.6 | 0.18 ± 0.051 | ARS-BFGL-NGS-29052, ARS-BFGL-NGS-59502, ARS-BFGL-NGS-101609 |  |  |
| 11 | 101.9 | 0.15 ± 0.047 | ARS-BFGL-NGS-118919 |  |  |
| 11 | 103 - 104.8 | 0.21 ± 0.05 | ARS-USMARC-Parent-AY851163-rs17871661, ARS-BFGL-NGS-26919, ARS-BFGL-NGS-98548, ARS-BFGL-NGS-9222, ARS-BFGL-NGS-114744, ARS-BFGL-NGS-77775, ARS-BFGL-NGS-39566, ARS-BFGL-NGS-104610, ARS-BFGL-NGS-100667 |  |  |
| 11 | 106 - 106.2 | 0.19 ± 0.053 | ARS-BFGL-NGS-82102, Hapmap42853-BTA-15475, ARS-BFGL-NGS-114194 |  |  |
| 12 | 22.7 - 22.9 | 0.18 ± 0.05 | Hapmap52315-rs29014466, ARS-BFGL-BAC-12458 |  |  |
| 12 | 24.7 - 27.3 | 0.18 ± 0.048 | ARS-BFGL-BAC-802, BTA-117635-no-rs, Hapmap48677-BTA-18532, Hapmap42159-BTA-26305, Hapmap49516-BTA-18533, BTA-120908-no-rs, BTA-18593-no-rs |  |  |
| 12 | 28.3 - 35.5 | 0.23 ± 0.047 | BTA-09514-rs29025674, ARS-BFGL-NGS-79502, ARS-BFGL-NGS-10225, Hapmap38930-BTA-100956, ARS-BFGL-BAC-15016, ARS-BFGL-BAC-15018, Hapmap42054-BTA-108170, BTA-18601-no-rs, ARS-BFGL-NGS-109579, ARS-BFGL-NGS-39585, ARS-BFGL-NGS-115095, BTA-20647-no-rs, ARS-BFGL-NGS-12, ARS-BFGL-NGS-23611, ARS-BFGL-NGS-31294, Hapmap48681-BTA-19661, ARS-BFGL-NGS-65358, ARS-BFGL-NGS-55763, ARS-BFGL-NGS-96494, ARS-BFGL-NGS-72624 |  |  |
| 12 | 36.7 - 37 | 0.17 ± 0.051 | Hapmap51939-BTA-21630, ARS-BFGL-NGS-82501 |  |  |
| 12 | 50.3 - 52 | 0.18 ± 0.05 | BTB-01236932, ARS-BFGL-NGS-116223, BTB-01834338, BTB-00494575, BTA-87640-no-rs, Hapmap51090-BTA-92858, BTB-01433297, BTB-00495782, BTB-00495684 |  |  |
| 12 | 59 | 0.15 ± 0.05 | BTB-00266282 |  |  |
| 12 | 61.2 - 62.3 | 0.19 ± 0.064 | BTB-00500246, BTB-01989473, BTA-85739-no-rs |  |  |
| 12 | 65.7 - 67.8 | 0.23 ± 0.049 | ARS-BFGL-NGS-10953, ARS-BFGL-BAC-14357, Hapmap57649-rs29022414, Hapmap46131-BTA-27009, Hapmap44023-BTA-122769, Hapmap28306-BTA-142098, BTB-00502495, BTB-00502512 |  |  |
| 12 | 69.1 - 70.8 | 0.23 ± 0.051 | ARS-BFGL-BAC-14990, Hapmap44078-BTA-45393, ARS-BFGL-NGS-3760, BTB-01626215 |  |  |
| 12 | 76.7 - 79.3 | 0.24 ± 0.051 | BTB-02095409, ARS-BFGL-NGS-4815, BTA-29081-no-rs, BTA-29998-no-rs, Hapmap58533-rs29018583, ARS-BFGL-NGS-44151, ARS-BFGL-NGS-115891, ARS-BFGL-NGS-117564 |  |  |
| 13 | 3.7 - 6.6 | 0.17 ± 0.05 | ARS-BFGL-NGS-37393, ARS-BFGL-NGS-57178, ARS-BFGL-NGS-104913, ARS-BFGL-NGS-103950, Hapmap25313-BTA-153969, BTB-01748916, BTB-00511781, BTB-01221558, Hapmap50223-BTA-113230, Hapmap35364-SCAFFOLD255028_58009, ARS-BFGL-NGS-84327, Hapmap54224-rs29017378 |  |  |
| 13 | 9 - 9.6 | 0.2 ± 0.049 | ARS-BFGL-NGS-966, Hapmap50661-BTA-33254, ARS-BFGL-NGS-16509, ARS-BFGL-NGS-116697, BTB-01135267, Hapmap53972-rs29024048, Hapmap55461-rs29025797, ARS-BFGL-NGS-19364, ARS-BFGL-NGS-119032 |  |  |
| 13 | 18 - 22.9 | 0.22 ± 0.053 | Hapmap55207-ss46526284, Hapmap51587-BTA-34220, Hapmap39556-BTA-34210, ARS-BFGL-NGS-21967, BTA-25900-no-rs, ARS-BFGL-NGS-112023, BTA-90722-no-rs, Hapmap51397-BTA-115996, ARS-BFGL-NGS-41168, Hapmap57706-rs29024881, ARS-BFGL-NGS-117417, ARS-BFGL-BAC-7467, ARS-BFGL-NGS-22974, ARS-BFGL-NGS-28633, ARS-BFGL-NGS-3658 |  |  |
| 13 | 24.4 - 25.3 | 0.13 ± 0.031 | ARS-BFGL-NGS-56387, ARS-BFGL-BAC-869, ARS-BFGL-NGS-15781, Hapmap43234-BTA-31907, ARS-BFGL-NGS-115560, ARS-BFGL-NGS-5327 |  |  |
| 13 | 26.8 | 0.06 ± 0.015 | BTA-13329-rs29018291 |  |  |
| 13 | 30.7 | 0.12 ± 0.031 | ARS-BFGL-NGS-55024 |  |  |
| 13 | 36.7 - 38.3 | 0.18 ± 0.04 | BTA-17784-no-rs, Hapmap51578-BTA-27320, BTA-32337-no-rs, BTB-00519631, ARS-BFGL-NGS-66410, BTA-32371-no-rs, ARS-BFGL-NGS-24450, BTB-00520031, ARS-BFGL-NGS-14560, ARS-BFGL-NGS-88501, ARS-BFGL-NGS-111603, ARS-BFGL-NGS-4298 |  |  |
| 13 | 55.8 - 56.8 | 0.19 ± 0.052 | ARS-BFGL-NGS-5166, ARS-BFGL-NGS-1365, Hapmap52629-rs29014811, ARS-BFGL-NGS-65704 |  |  |
| 13 | 58.2 - 59.2 | 0.13 ± 0.038 | ARS-BFGL-NGS-38704, ARS-BFGL-NGS-352 |  |  |
| 13 | 62.9 | 0.12 ± 0.036 | ARS-BFGL-NGS-56157 |  |  |
| 13 | 65 - 65.6 | 0.13 ± 0.035 | ARS-BFGL-BAC-847, BTB-00537534 |  |  |
| 13 | 69.7 - 70.6 | 0.18 ± 0.044 | ARS-BFGL-NGS-103010, ARS-BFGL-NGS-106716, ARS-BFGL-NGS-112608, Hapmap25516-BTA-128677, ARS-BFGL-NGS-112889 |  |  |
| 13 | 72.1 - 75.6 | 0.16 ± 0.038 | ARS-BFGL-NGS-38677, ARS-BFGL-NGS-108803, ARS-BFGL-NGS-71538, ARS-BFGL-NGS-97229, ARS-BFGL-NGS-117163, ARS-BFGL-NGS-114029, ARS-BFGL-NGS-4602, ARS-BFGL-NGS-116438, ARS-BFGL-NGS-43096, ARS-BFGL-NGS-89241, ARS-BFGL-NGS-112625, Hapmap31079-BTA-128662, BTB-00750399, ARS-BFGL-NGS-30791, BTA-33806-no-rs |  |  |
| 13 | 77 | 0.09 ± 0.022 | ARS-BFGL-NGS-96358 |  |  |
| 13 | 78.6 | 0.16 ± 0.05 | ARS-BFGL-NGS-41546 |  |  |
| 13 | 80.5 - 81.8 | 0.17 ± 0.047 | ARS-BFGL-NGS-82178, ARS-BFGL-NGS-112197, ARS-BFGL-NGS-118584, ARS-BFGL-NGS-47826, Hapmap53515-rs29024560, UA-IFASA-4008, Hapmap61120-rs29012586 |  |  |
| 14 | 16.2 | 0.07 ± 0.023 | ARS-BFGL-NGS-101433 |  |  |
| 14 | 17.5 | 0.07 ± 0.023 | ARS-BFGL-NGS-82859 |  |  |
| 14 | 19.8 - 23.6 | 0.15 ± 0.045 | BTA-18878-no-rs, ARS-BFGL-NGS-117284, Hapmap35443-SCAFFOLD20068_27016, ARS-BFGL-NGS-36955, UA-IFASA-7482, BTB-01251993, ARS-BFGL-BAC-12159, BTB-00556983, Hapmap49966-BTA-34362 | PWG, CW | Mizoshita et al., 2005; Kneeland et al., 2004; Takasuga et al., 2007 |
| 14 | 29.1 | 0.13 ± 0.04 | UA-IFASA-8638, UA-IFASA-9439 |  |  |
| 14 | 36.3 | 0.09 ± 0.031 | BTB-00564573 |  |  |
| 14 | 37.8 - 38.4 | 0.06 ± 0.018 | ARS-BFGL-NGS-36094, BTB-01190209, BTA-107899-no-rs |  |  |
| 14 | 44.1 | 0.06 ± 0.018 | ARS-BFGL-BAC-21453 |  |  |
| 14 | 74 - 74.6 | 0.19 ± 0.043 | ARS-BFGL-NGS-12514, UA-IFASA-8614, Hapmap22793-BTA-129434, Hapmap58449-rs29021974, UA-IFASA-9661 |  |  |
| 15 | 5.9 | 0.17 ± 0.051 | BTA-55622-no-rs |  |  |
| 15 | 9.5 | 0.15 ± 0.048 | BTB-01417458 |  |  |
| 15 | 27.1 - 27.9 | 0.08 ± 0.027 | Hapmap39993-BTA-36164, ARS-BFGL-NGS-31970 |  |  |
| 15 | 31.6 | 0.06 ± 0.015 | ARS-BFGL-NGS-100678 |  |  |
| 15 | 38 - 39.9 | 0.19 ± 0.053 | ARS-BFGL-NGS-115263, Hapmap43562-BTA-36560, Hapmap50456-BTA-72525, Hapmap47272-BTA-36655, ARS-BFGL-NGS-118296, Hapmap57927-rs29025966, ARS-BFGL-NGS-110928 | ADG | Marquez et al., 2009 |
| 15 | 41.4 - 46 | 0.17 ± 0.051 | ARS-BFGL-NGS-119103, Hapmap50841-BTA-99608, ARS-BFGL-NGS-73907, ARS-BFGL-NGS-31299, ARS-BFGL-NGS-32123, ARS-BFGL-NGS-25667, ARS-BFGL-NGS-81500, BTB-00596891, BTB-02091993, BTB-01120372, ARS-BFGL-NGS-39267, ARS-BFGL-NGS-22122 |  |  |
| 15 | 50.2 | 0.15 ± 0.049 | ARS-BFGL-NGS-102432 |  |  |
| 15 | 62 - 65.3 | 0.22 ± 0.049 | ARS-BFGL-NGS-41462, Hapmap25081-BTA-153963, BTB-01177498, BTB-01778967, BTB-01177481, BTB-01177461, ARS-BFGL-NGS-77090, BTB-01230622, ARS-BFGL-NGS-112293, BTB-01788867, ARS-BFGL-NGS-63905, BTB-01508301, ARS-BFGL-NGS-82738, BTA-18105-no-rs, Hapmap48636-BTA-118794, Hapmap59484-rs29019823, BTB-01505484, ARS-BFGL-NGS-43586, BTB-00610177, BTA-37293-no-rs |  |  |
| 16 | 0.1 | 0.19 ± 0.051 | ARS-BFGL-NGS-66185 |  |  |
| 16 | 1.6 - 4 | 0.2 ± 0.051 | ARS-BFGL-NGS-97259, ARS-BFGL-NGS-110956, ARS-BFGL-NGS-10342, BTB-00626834, ARS-BFGL-NGS-117331, ARS-BFGL-NGS-22732, BTB-01175421, BTB-00624204, BTA-38126-no-rs |  |  |
| 16 | 5.1 | 0.15 ± 0.05 | ARS-BFGL-NGS-118841 |  |  |
| 16 | 16.5 | 0.07 ± 0.022 | Hapmap42889-BTA-27057 |  |  |
| 16 | 64 - 70.2 | 0.21 ± 0.048 | ARS-BFGL-NGS-89882, BTB-01547185, Hapmap52108-ss46526959, ARS-BFGL-NGS-10682, ARS-BFGL-NGS-103362, ARS-BFGL-NGS-33008, BTB-01650065, ARS-BFGL-NGS-117996, ARS-BFGL-NGS-113169, ARS-BFGL-NGS-45685, ARS-BFGL-NGS-62668, BTA-26576-no-rs, BTB-01346111, ARS-BFGL-NGS-101250, Hapmap59847-rs29025534, BTB-00654373, BTB-00656287, BTB-00656184, BTB-00656135, BTA-39826-no-rs, BTA-107407-no-rs, BTA-39836-no-rs, ARS-BFGL-NGS-81253, Hapmap40306-BTA-39850, Hapmap39462-BTA-111304, BTA-111303-no-rs, ARS-BFGL-NGS-30006, BTA-90680-no-rs, BTB-01660545, Hapmap33514-BTA-158988, Hapmap42075-BTA-114094, BTB-01188142, BTB-01188274, Hapmap51136-BTA-107420 |  |  |
| 16 | 72.1 - 72.6 | 0.15 ± 0.049 | ARS-BFGL-NGS-17251, Hapmap53215-rs29019312 |  |  |
| 16 | 74 - 74.3 | 0.17 ± 0.048 | Hapmap51565-BTA-122868, ARS-BFGL-NGS-20274 |  |  |
| 16 | 79.9 | 0.09 ± 0.03 | ARS-BFGL-NGS-36059 |  |  |
| 17 | 10.5 | 0.13 ± 0.042 | BTB-00671090 |  |  |
| 17 | 11.8 - 15.3 | 0.16 ± 0.052 | BTB-00671689, BTB-00671736, Hapmap52851-rs29021975, BTB-00675011, Hapmap57405-rs29021707, BTA-19257-no-rs, BTA-42221-no-rs, BTA-05721-rs29019877 |  |  |
| 17 | 18.4 - 20.3 | 0.14 ± 0.046 | ARS-BFGL-NGS-11296, ARS-BFGL-NGS-116291, BTA-25641-no-rs, Hapmap31828-BTA-159336, ARS-BFGL-NGS-25537, BTA-16045-no-rs, Hapmap48663-BTA-16043, Hapmap48748-BTA-40639, ARS-BFGL-NGS-110834 |  |  |
| 17 | 21.5 - 23.7 | 0.17 ± 0.047 | Hapmap58447-rs29021883, ARS-BFGL-BAC-35973, ARS-BFGL-NGS-107447, ARS-BFGL-NGS-45739, Hapmap55890-rs29027310, ARS-BFGL-BAC-2274, Hapmap43260-BTA-40679, Hapmap52011-BTA-40675, BTA-40659-no-rs, Hapmap36431-SCAFFOLD200017_11121, ARS-BFGL-BAC-35971, ARS-BFGL-NGS-25840 |  |  |
| 17 | 24.8 - 27 | 0.16 ± 0.036 | ARS-BFGL-BAC-4867, Hapmap33840-BES7_Contig516_781, Hapmap39152-BTA-27712, ARS-BFGL-BAC-18831, Hapmap51732-BTA-46639, BTA-46650-no-rs, Hapmap34820-BES2_Contig369_543, Hapmap43860-BTA-46675 |  |  |
| 17 | 28.2 - 28.3 | 0.11 ± 0.028 | Hapmap55423-rs29013623, ARS-BFGL-NGS-68295, Hapmap35289-BES4_Contig230_1042 |  |  |
| 17 | 30.7 - 32.1 | 0.11 ± 0.024 | ARS-BFGL-NGS-61134, BTB-01869986, ARS-BFGL-NGS-96366, BTA-104167-no-rs, BTA-102388-no-rs, BTB-01393971 |  |  |
| 17 | 69.1 - 72.2 | 0.16 ± 0.042 | BTB-01121732, ARS-BFGL-BAC-35593, BTB-01130358, BTB-01682471, ARS-BFGL-NGS-93586, Hapmap47002-BTA-41821, ARS-BFGL-NGS-1166, ARS-BFGL-NGS-62271, ARS-BFGL-NGS-69511, Hapmap29812-BTA-131753, ARS-BFGL-NGS-70175, ARS-BFGL-NGS-36744, Hapmap53106-rs29017719, ARS-BFGL-NGS-102559 |  |  |
| 18 | 6.4 - 6.9 | 0.1 ± 0.028 | ARS-BFGL-NGS-106794, ARS-BFGL-NGS-58546, ARS-BFGL-NGS-109552 |  |  |
| 18 | 8 | 0.15 ± 0.046 | ARS-BFGL-NGS-73708 |  |  |
| 18 | 14.4 - 14.5 | 0.1 ± 0.029 | Hapmap44238-BTA-42338, ARS-BFGL-NGS-24837, ARS-BFGL-NGS-13803 |  |  |
| 18 | 16.7 - 17.5 | 0.19 ± 0.049 | ARS-BFGL-NGS-24006, ARS-BFGL-NGS-55981, ARS-BFGL-NGS-109919, ARS-BFGL-NGS-81231, ARS-BFGL-NGS-89344, ARS-BFGL-NGS-107392, Hapmap39998-BTA-42612 |  |  |
| 18 | 19.2 | 0.07 ± 0.019 | ARS-BFGL-NGS-111479 |  |  |
| 18 | 35.6 - 36 | 0.16 ± 0.047 | Hapmap34397-BES7_Contig335_1167, ARS-BFGL-NGS-113568 |  |  |
| 18 | 37.3 - 40.5 | 0.16 ± 0.042 | Hapmap49605-BTA-43064, BTA-43104-no-rs, BTA-43160-no-rs, Hapmap58822-rs29018153, ARS-BFGL-NGS-96821, ARS-BFGL-NGS-108025, ARS-BFGL-NGS-117852, ARS-BFGL-NGS-101288, ARS-BFGL-NGS-113001, ARS-BFGL-NGS-101743, ARS-BFGL-NGS-79228 | DMI | Nkrumah et al.2007 |
| 18 | 55.8 - 55.9 | 0.15 ± 0.047 | ARS-BFGL-NGS-93837, ARS-BFGL-NGS-40328 |  |  |
| 18 | 65.5 - 65.6 | 0.12 ± 0.032 | ARS-BFGL-NGS-60589, ARS-BFGL-NGS-58016 |  |  |
| 19 | 15.2 - 17.9 | 0.2 ± 0.053 | ARS-BFGL-NGS-52513, ARS-BFGL-NGS-118126, ARS-BFGL-NGS-44497, ARS-BFGL-NGS-26149, ARS-BFGL-NGS-115774, Hapmap51231-BTA-44563, ARS-BFGL-NGS-42338, ARS-BFGL-NGS-105643, ARS-BFGL-NGS-58978, ARS-BFGL-NGS-19462, ARS-BFGL-NGS-26897 |  |  |
| 19 | 36.5 | 0.11 ± 0.034 | ARS-BFGL-NGS-114136 |  |  |
| 19 | 41.5 - 41.6 | 0.12 ± 0.035 | Hapmap54523-ss46526232, UA-IFASA-6957 |  |  |
| 19 | 61.5 - 61.9 | 0.06 ± 0.02 | ARS-BFGL-NGS-116261, Hapmap43271-BTA-46356, ARS-BFGL-NGS-103044 |  |  |
| 19 | 64 | 0.05 ± 0.016 | ARS-BFGL-NGS-119772 |  |  |
| 20 | 2.6 - 3.1 | 0.2 ± 0.05 | ARS-BFGL-NGS-25164, BTB-01393816, ARS-BFGL-NGS-114238 |  |  |
| 20 | 8.5 - 9.1 | 0.14 ± 0.046 | BTB-00771346, BTB-01482697, BTA-51491-no-rs |  |  |
| 20 | 11.4 - 12.2 | 0.06 ± 0.019 | Hapmap46331-BTA-96019, Hapmap42086-BTA-118249 |  |  |
| 20 | 46.4 - 46.8 | 0.14 ± 0.045 | BTA-50702-no-rs, BTB-01376724, Hapmap35236-BES3_Contig374_812 |  |  |
| 20 | 63.8 | 0.13 ± 0.041 | Hapmap38992-BTA-96283 |  |  |
| 20 | 65.5 | 0.11 ± 0.034 | ARS-BFGL-NGS-117373 |  |  |
| 20 | 66.7 - 67.1 | 0.13 ± 0.043 | ARS-BFGL-NGS-22607, ARS-BFGL-NGS-7347 |  |  |
| 20 | 68.3 | 0.11 ± 0.031 | ARS-BFGL-NGS-2865 |  |  |
| 20 | 69.3 | 0.11 ± 0.03 | ARS-BFGL-NGS-14372 |  |  |
| 21 | 11.2 | 0.16 ± 0.051 | BTB-01718760 |  |  |
| 21 | 15.7 | 0.14 ± 0.044 | BTB-00222111 |  |  |
| 21 | 52.2 - 54.2 | 0.23 ± 0.047 | ARS-BFGL-NGS-67889, Hapmap50580-BTA-116485, BTA-116497-no-rs, BTB-00649157, BTB-01814935, BTB-00824019, BTB-01046504, BTB-01046586, ARS-BFGL-NGS-46793 |  |  |
| 21 | 55.5 | 0.14 ± 0.04 | BTA-98184-no-rs |  |  |
| 21 | 65.5 - 67.2 | 0.17 ± 0.044 | Hapmap54413-rs29026873, BTA-52926-no-rs, ARS-BFGL-NGS-37574, Hapmap35472-SCAFFOLD35342_21388, ARS-BFGL-NGS-100337, Hapmap35139-BES10_Contig553_546, ARS-BFGL-NGS-113795 |  |  |
| 22 | 0.8 | 0.16 ± 0.044 | ARS-BFGL-NGS-114733 |  |  |
| 22 | 2.6 - 3 | 0.18 ± 0.052 | Hapmap41765-BTA-119173, Hapmap47324-BTA-55159, ARS-BFGL-NGS-30499 |  |  |
| 22 | 5.4 - 6.9 | 0.15 ± 0.05 | BTA-54457-no-rs, ARS-BFGL-NGS-4304, Hapmap60747-rs29010034, Hapmap58102-rs29011445, ARS-BFGL-NGS-66672, BTB-00832995, ARS-BFGL-NGS-118232 |  |  |
| 22 | 26.5 | 0.18 ± 0.055 | ARS-BFGL-BAC-35183, Hapmap43510-BTA-20951 |  |  |
| 22 | 42.3 | 0.08 ± 0.025 | ARS-BFGL-NGS-110255 |  |  |
| 22 | 59.6 - 59.7 | 0.09 ± 0.028 | ARS-BFGL-NGS-24884, ARS-BFGL-NGS-54563 |  |  |
| 22 | 60.9 | 0.09 ± 0.029 | ARS-BFGL-NGS-33950, ARS-BFGL-NGS-30266 |  |  |
| 23 | 19.2 - 20.2 | 0.16 ± 0.045 | UA-IFASA-1570, BTA-55757-no-rs, ARS-BFGL-NGS-111955, ARS-BFGL-NGS-16397, ARS-BFGL-BAC-29490 | GBW, HW | Elo et al., 1999; Kneeland et al., 2004 |
| 23 | 21.8 | 0.15 ± 0.05 | Hapmap35058-BES7_Contig424_1473 |  |  |
| 23 | 23 - 24.5 | 0.19 ± 0.049 | ARS-BFGL-BAC-6039, ARS-BFGL-NGS-114061, Hapmap44335-BTA-106037, Hapmap47327-BTA-55967 |  |  |
| 23 | 40.4 | 0.16 ± 0.049 | ARS-BFGL-BAC-46684, Hapmap31542-BTA-56774 |  |  |
| 23 | 48.7 - 49 | 0.1 ± 0.033 | ARS-BFGL-NGS-32006, ARS-BFGL-NGS-36409 | DMI | Sherman et al., 2009 |
| 23 | 50.4 - 51.2 | 0.1 ± 0.034 | ARS-BFGL-NGS-11502, ARS-BFGL-NGS-108494 |  |  |
| 24 | 1.5 | 0.16 ± 0.051 | Hapmap33874-BES10_Contig434_1045 |  |  |
| 24 | 2.8 | 0.15 ± 0.05 | Hapmap48003-BTA-57985 |  |  |
| 24 | 8.4 - 9.1 | 0.08 ± 0.022 | ARS-BFGL-BAC-37052, BTA-115451-no-rs, Hapmap38789-BTA-86678 |  |  |
| 24 | 11.1 - 11.7 | 0.16 ± 0.048 | BTB-00881336, BTB-00881459, Hapmap48813-BTA-57373, BTB-00882425 |  |  |
| 24 | 15.1 | 0.14 ± 0.045 | ARS-BFGL-NGS-20239 |  |  |
| 24 | 18.7 | 0.14 ± 0.047 | Hapmap42090-BTA-118962 |  |  |
| 24 | 24.2 | 0.05 ± 0.018 | Hapmap38596-BTA-41284 |  |  |
| 24 | 27.4 - 29.2 | 0.12 ± 0.032 | Hapmap60145-rs29013637, Hapmap49102-BTA-27356, BTA-57747-no-rs, Hapmap54558-rs29009598 |  |  |
| 24 | 55.5 - 56.9 | 0.07 ± 0.023 | ARS-BFGL-NGS-65432, Hapmap51262-BTA-58394, Hapmap59021-rs29022078 |  |  |
| 25 | 5.2 - 5.3 | 0.15 ± 0.046 | Hapmap26242-BTC-001240, Hapmap25382-BTC-000577 |  |  |
| 25 | 7.6 | 0.14 ± 0.048 | ARS-BFGL-NGS-90194 |  |  |
| 25 | 11.2 - 11.4 | 0.16 ± 0.049 | Hapmap32921-BTC-023401, Hapmap26623-BTC-073724 |  |  |
| 25 | 12.7 - 13.9 | 0.15 ± 0.047 | BTB-00898931, Hapmap23896-BTA-59579, Hapmap52158-rs29019621, Hapmap40768-BTA-59535 |  |  |
| 25 | 16.9 | 0.13 ± 0.036 | Hapmap57057-rs29022935 |  |  |
| 26 | 20.4 | 0.17 ± 0.05 | ARS-BFGL-NGS-23064 |  |  |
| 26 | 27.9 - 32.5 | 0.19 ± 0.049 | Hapmap29376-BTA-72268, ARS-BFGL-NGS-62893, Hapmap38478-BTA-20824, ARS-BFGL-NGS-20025, BTA-107261-no-rs, ARS-BFGL-NGS-109588, Hapmap23820-BTA-149582, ARS-BFGL-NGS-32374, BTB-00937875, Hapmap38330-BTA-61119, Hapmap47348-BTA-61100, Hapmap48021-BTA-61134, BTB-00938457 | ADG | Gutierrez-Gil et al., 2009 |
| 26 | 35.4 | 0.05 ± 0.016 | Hapmap42605-BTA-61330 |  |  |
| 26 | 37.4 - 38.3 | 0.07 ± 0.021 | BTA-61460-no-rs, BTA-05179-no-rs, BTA-10577-no-rs |  |  |
| 26 | 40.6 | 0.14 ± 0.044 | BTA-100861-no-rs |  |  |
| 26 | 42.2 | 0.1 ± 0.032 | ARS-BFGL-NGS-5157 |  |  |
| 26 | 44.3 | 0.13 ± 0.042 | ARS-BFGL-NGS-36401 |  |  |
| 26 | 45.5 - 47.9 | 0.15 ± 0.049 | Hapmap41037-BTA-61760, ARS-BFGL-NGS-44396, ARS-BFGL-NGS-112732, ARS-BFGL-NGS-74523, ARS-BFGL-NGS-32517 | ADG, DMI | Nkrumah et al.2007 |
| 26 | 50.8 - 51 | 0.18 ± 0.054 | ARS-BFGL-NGS-86889, ARS-BFGL-NGS-76872 |  |  |
| 27 | 27.9 | 0.12 ± 0.038 | BTB-01064330 |  |  |
| 27 | 29.1 - 30.1 | 0.15 ± 0.038 | BTA-97696-no-rs, Hapmap53111-rs29024461, ARS-BFGL-NGS-53829 |  |  |
| 27 | 31.5 | 0.15 ± 0.044 | Hapmap40631-BTA-103396 |  |  |
| 27 | 32.5 - 32.8 | 0.06 ± 0.017 | ARS-BFGL-NGS-112547, ARS-BFGL-NGS-73189 |  |  |
| 27 | 36.3 - 37.5 | 0.05 ± 0.014 | ARS-BFGL-NGS-88722, ARS-BFGL-NGS-24680, ARS-BFGL-NGS-4687 |  |  |
| 28 | 6 | 0.08 ± 0.025 | ARS-BFGL-NGS-111192 |  |  |
| 28 | 42 | 0.16 ± 0.05 | BTA-99379-no-rs |  |  |
| 28 | 45.9 - 46 | 0.13 ± 0.041 | ARS-BFGL-NGS-119491, BTB-00995363 |  |  |
| 29 | 0.6 - 2.2 | 0.16 ± 0.045 | Hapmap53898-rs29026969, Hapmap39498-BTA-66465, Hapmap43008-BTA-65226, ARS-BFGL-NGS-17668, ARS-BFGL-NGS-43123, Hapmap35197-BES11_Contig441_882, ARS-BFGL-NGS-119674 |  |  |
| 29 | 3.2 | 0.24 ± 0.068 | ARS-BFGL-NGS-90518 |  |  |
| 29 | 11.3 | 0.06 ± 0.021 | ARS-BFGL-NGS-41836 |  |  |
| 29 | 29 - 29.3 | 0.13 ± 0.044 | ARS-BFGL-NGS-118467, ARS-BFGL-NGS-39174 |  |  |

^1^ The maximum regression coefficient for number of SNP minor alleles (0, 1, 2) at a locus on CSD (kg×100) for postweaning gain.

^2^ SNP wherein the regression (b ± SE) of number of minor alleles on cumulative selection differential for postweaning gain was significant (FDR < 0.05) in L1

^3^ Previously identified QTL for traits: ADG = Average daily gain, CW = Carcass weight, DMI = dry matter intake, PWG = Postweaning gain, GBW = Preweaning gain, HW = Harvest weight, YW = 365-d weight.
